# The consolidation of nanomedicine

**DOI:** 10.1002/wnan.1569

**Published:** 2019-06-26

**Authors:** Raphael Zingg, Marius Fischer

**Affiliations:** ^1^ Institute for Advanced Study Waseda University Tokyo Japan; ^2^ Center for Law & Economics ETH Zurich Zurich; ^3^ Max Planck Institute for Innovation and Competition Munich Germany

**Keywords:** nanomedicine innovation, network analysis, patent citations, regulatory uncertainty

## Abstract

Over the past two decades, nanomedicine has grown steadily, however, without inducing a palpable shift in the diagnosis and treatment of diseases so far. While this may simply be a consequence of the slow, incremental nature that characterizes many modern technologies, this article posits that there is another set of significant factors harboring explanatory power. Uncertainties concerning safety, regulatory, and ethical requirements may have prompted innovators to stay close to the known and approved, eventually at the cost of innovating in unexplored alleys. Network analysis of all nanomedicine patents in the United States reveals that nanomedicine has indeed rather consolidated than expanded. We detail a set of recommendations that would reduce the uncertainty prevailing in nanomedicine and could contribute to pushing new boundaries.

This article is categorized under:

Toxicology and Regulatory Issues in Nanomedicine > Regulatory and Policy Issues in Nanomedicine

## Graphical abstract

Despite being a broad field at the frontier of the life sciences, innovation in nanomedicine seems to have rather consolidated than expanded. Uncertainties in safety, regulatory and ethical requirements may have led innovators to engage in nanomedicine that relies on past innovation, eventually at the expense of fostering advances in novel fields.

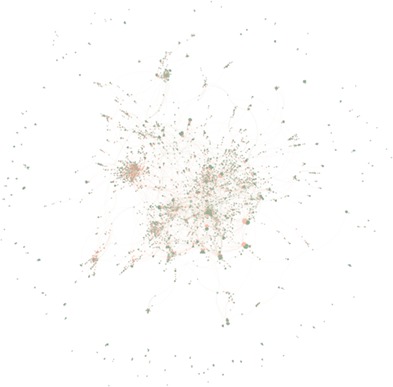

## 1 INTRODUCTION

Ever since the U.S. Food and Drug Administration (FDA) approved a nanotherapeutic product for the first time back in 1995—Doxil, an anticancer drug using PEGylated nano‐liposomes—the dawn of nanomedicine has been proclaimed. In fact, however, despite a number of clinical successes that have impacted cardiology and oncology in particular, nanotechnology has not yet revolutionized the diagnosis and treatment of diseases on a large scale. For pharmaceuticals, in vivo imaging and in vitro diagnostics, nanotechnology is poised to contribute significantly, but the technology has yet to bear its fruits. Many argue that this results at least partly from the uncertainties regarding the health risks nanomaterials may pose (Agarwal, Bajpai, & Sharma, 2018; Jongandand & Borm, 2008). While this is a problem for nanotechnology as a whole, the legal regulation of nanomedicine introduces a special set of constraints worth an independent study (Bregoli et al., 2016; Burgess et al., 2010). Safety concerns, relating to nanomaterial‐induced toxicity effects in particular, have nanopharmaceuticals go through more elaborate and more costly testings (Venkatraman, 2014). The lack of validated methods for toxicity testing assays and the limited understanding of the interaction of nanomaterials with biological systems complicate the establishment of accepted health risk assessments (Halappanavar, Vogel, Wallin, & Yauk, 2018). From a regulatory standpoint, the FDA has failed to set forth practical assays, testing, or data requirements (Bawa, Barenholz, & Owen, 2016). The resulting uncertainty may explain nanomedicine's underperformance at clinical trial stage, a primary reason for its slow translation into approved therapeutic therapies (Weissig & Guzman‐Villanueva, 2015; Yu & Bae, 2018). In December 2017, the agency finally released a non‐binding draft guidance for industry in order to address the safety and efficacy challenges of approving complex drugs containing nanomaterials.^1^ Still, this leaves room for a number of uncertainties concerning possible approval pathways (Emily, Ioanna, Scott, & Beat, 2018).

This article advances that, in light of these uncertainties, nanomedicine consolidated rather than expanded. In short, we contend that innovators mostly sought to deal with the uncertainties inherent to nanomedicine by trying to build upon approved technologies. Typically, firms would favor scientifically crowded fields where other nanomedicine inventors already paved the way. Accordingly, the industry would seek to tailor nano‐enabled products to known markets rather than to explore the technology's potential to its full extent (Bosetti & Vereeck, 2012; Neuman & Chandhok, 2016; Pelaz et al., 2017). While we realize that there undoubtedly are advantages to leveraging closely related research to leap forward, that is, to stand on the shoulders of giants, we contend that too close an adherence to past research might have prevented nanomedicine from diving into unexplored fields. We believe this phenomenon may, for example, be observed with respect to advances—or lack thereof—in nanopharmaceuticals. Of the 50 nanopharmaceuticals that had received FDA approval in 2016, almost all were nanoformulations of existing drugs rather than providing a novel pharmacological effect themselves. Their clinical benefits have been mainly limited to reductions in toxicity rather than improvements in efficacy, not fully realizing the high expectations of the scientific and medical community (Joseph, Artish, Tian, & Andrew, 2017; Ventola, 2012). Despite being commercial successes, Doxil did not revolutionize chemotherapy, nor did other nanocarrier therapeutics such as Abraxane and Ambiosome or nanorcystalline drugs such as Rapamune (Venkatraman, 2014). To fully turn the potential of nanopharmaceuticals into clinical formulations, additional advances in, and deeper understanding of, drug‐loading capacities, drug‐release control, clearance or degradation at target site, cellular uptake, and interaction with biological systems are needed (Nassiri & Abdollahi, 2016; Park, 2013; Qiao et al., 2019).

## 2 THE EMERGENCE OF NANOMEDICINE

**Figure 1:**
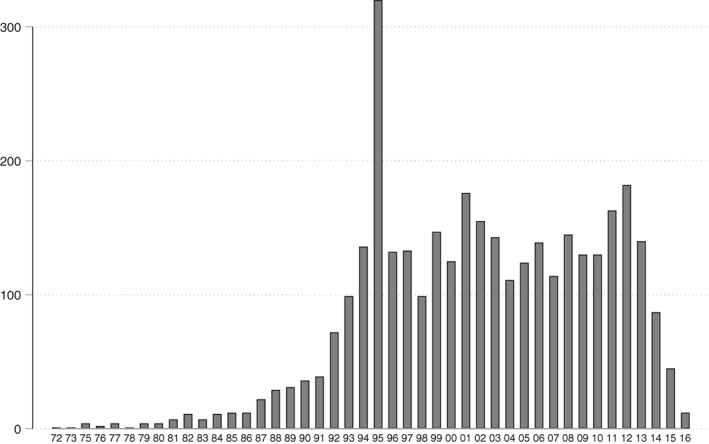
Evolution of US patents in nanomedicine (by year of filing 1972–2016)

Nanomedicine patents granted in the United States can be used to investigate this claim. Patent data can be extracted using PatentsView, a database sourced from United States Patent and Trademark Office provided data. Nanomedicine patents are classified together with nanobiotechnology in class B82Y 5/00 of the International Patent Classification (Jürgens & Herrero‐Solana, 2017).^2^ While patents granted for nanomedicine inventions have been on the rise, the numbers are still quite low. The annual rate of filing stabilized at around 150 applications a year since 1996 (see Figure 1). Note that the most recent statistics are incomplete since patent applications are published 18 months after their earliest filing date only.

Overall, nanomedicine patents thus only represent 3,497 out of the total 48,212 patents granted in nanotechnology (1974–2016). When filing for a patent, all applicants, their attorneys and agents have a duty to disclose all information known to be material to patentability, other patents in particular (Title 37 of the Code of Federal Regulations, §1.56[a]). Failure to comply bears drastic consequences: a patent may be declared unenforceable for its term (Erstling, 2011). Therefore, citations serve as a good indicator for the prior art that was relied on when inventing (Barirani, Agard, & Beaudry, 2013). Corresponding information was extracted from PatentsView, as well. Through this, we gain an overview of the emergence of nanomedicine, that is, what prior art it built upon over time. Note that patents cited by nanomedicine patents may not necessarily pertain to nanomedicine themselves.

## 3 THE CONSOLIDATION OF NANOMEDICINE

**Figure 2:**
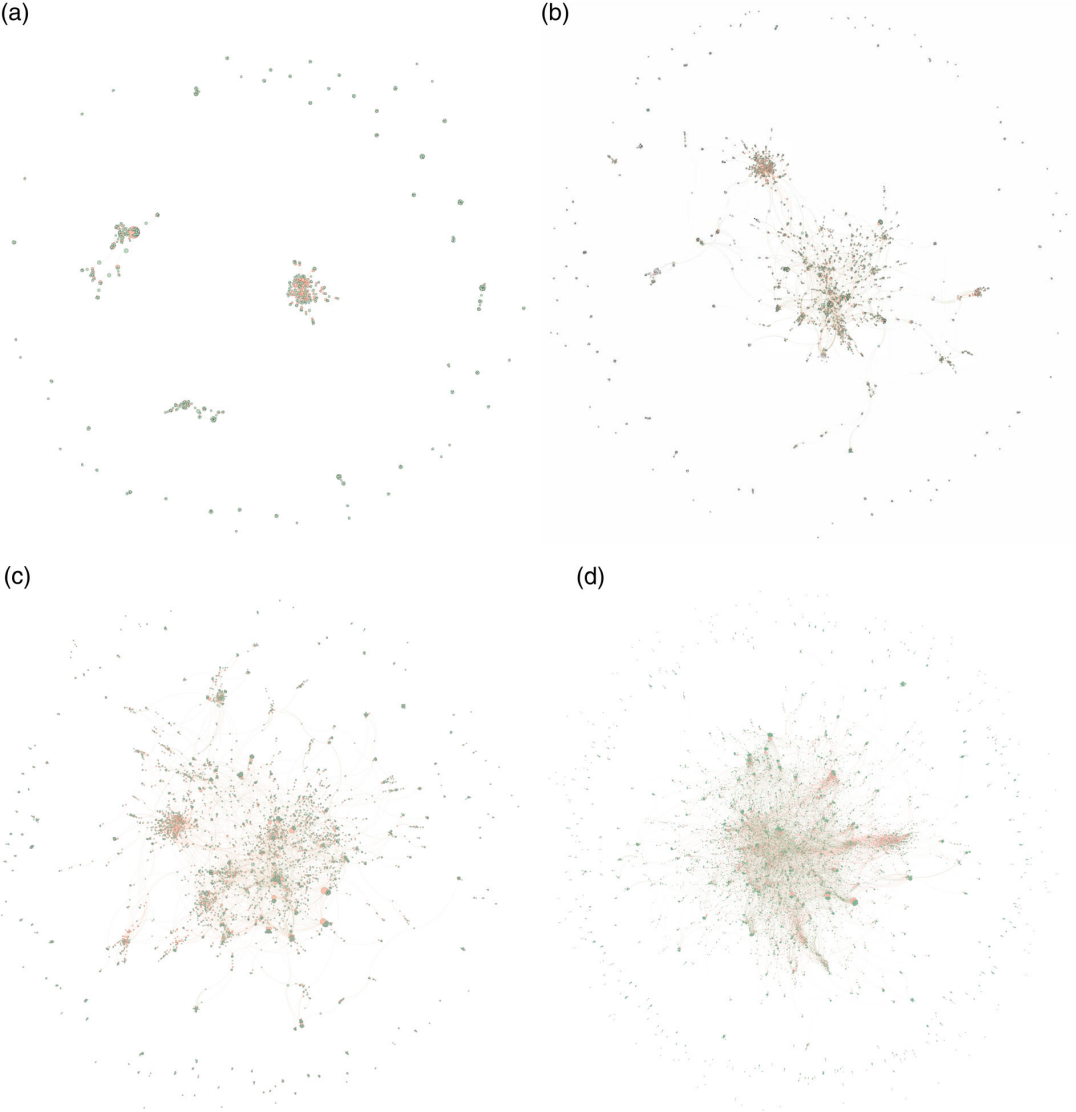
Network of nanomedicine patents prior art. (a) 1974–1990; (b) 1974–1995; (c) 1974–2000; (d) 1974–2016. The networks depict nanomedicine patents and their cited patents as nodes. Edges are established by citations. (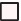) Nanomedicine patents; (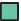) non‐nanomedicine patents

Figure 2 depicts the evolution of nanomedicine in terms of patent citation networks. Therein, nodes represent patents, and links between nodes are established when one of the patents cites the other. When examining these networks of patent citations, one can observe a trend toward consolidation. In the early stages from 1974 to 1990, three small “islands” of prior art had formed in isolation. Then they bridged in 1995. From then on, nanomedicine grew increasingly closer. Nowadays, despite spanning a variety of areas, almost all patents are linked to each other either directly or indirectly through common prior art. Furthermore, although the vast majority of cited patents are non‐nanomedicinal (88%), a majority of patents refers to at least one nanomedicine patent (up to 80% in 2014). Rather than branching out in specialized subfields, nanomedicine has been increasingly consolidating.

In fact, the central nodes in the networks are mostly nanomedicinal: Out of the 10 most frequently cited patents, seven are classified as nanomedicine. Four of these patents relate to mixing essentially water‐insoluble drugs with cyclodextrin—molecules with a size of about 1 nm—in order to obtain amorphous complexes that are themselves in turn highly water‐soluble (Patents 1, 4, 7, and 8 according to Table 1). In other words, they pertain to new formulations of known drugs that significantly enhance their absorption by the human body. Patent 3 according to Table 1 relates to using surface modifiers in order to keep an average particle diameter of a drug below 400 nm, vastly improving the drug's bioavailability. Patent 5 addresses selective targeting of radionuclides to solid tumor areas within the body. Similarly, Patent 6 pertains to providing block copolymers that can be used for controlled delivery of biologically active materials. As apparent from this analysis, the building blocks of nanomedicine indeed seem to be inventions covering new formulations of known active substances rather than independent, new products.

**Table 1:** Top 10 most cited patents in nanomedicine

	Patent number	Year	Technology	Title	Citations
1	US4727064	1993	Nanomedicine	Pharmaceutical preparations containing cyclodextrin derivatives	140
2	US5270163	1997	Other	Methods for identifying nucleic acid ligands	91
3	US5145684	2007	Nanomedicine	Surface modified drug nanoparticles	63
4	US4596795	1985	Nanomedicine	Administration of sex hormones in the form of hydrophilic cyclodextrin derivatives	60
5	US4863713	1993	Nanomedicine	Method and system for administering therapeutic and diagnostic agents	60
6	US5543158	2004	Nanomedicine	Biodegradable injectable nanoparticles	57
7	US5024998	2001	Nanomedicine	Pharmaceutical formulations for parenteral use	55
8	US4983586	1990	Nanomedicine	Pharmaceutical formulations for parenteral use	53
9	US5256395	2010	Other	Affinity enhancement immunological reagents for in vivo detection and killing of specific target cells	50
10	US5143854	2011	Other	Large scale photolithographic solid phase synthesis of polypeptides and receptor binding screening thereof	48

## 4 PUSHING THE BOUNDARIES IN NANOMEDICINE

Despite being such a broad and interdisciplinary field at the frontier of the life sciences, research in nanomedicine so far seems to have rather consolidated than expanded. It may have been uncertainties in safety, regulatory and ethical requirements that have led innovators to engage in nanomedicine that relies on past nanomedical innovation, eventually at the expense of fostering advances in novel fields. To reduce said uncertainty, a number of steps could be taken. Firstly, federal and international regulatory agencies should start by establishing regulatory definitions or common working descriptions of key terms like “nanotechnology,” “nanomaterial,” and “nanomedicine” to ensure harmonized governance (Bartlett et al., 2015; Bawa et al., 2016; Pita, Ehmann, & Papaluca, 2016; Tinkle et al., 2014). Secondly, regulatory bodies should seek to move from a non‐binding draft recommendation regime to a definite and enforceable one. Current regulations in nanomedicine comprise only a body of reflection papers in Europe and Japan, and industry guidelines in Canada and the United States (Bremer‐Hoffmann, Halamoda‐Kenzaoui, & Borgos, 2018). Specific regulations and protocols for preclinical development and characterization would provide stakeholders with the certainty they seek (Bawa et al., 2016; Marchant & Abbott, 2013; Sainz et al., 2015). Thirdly, to counter the lack of product safety data, the agencies should develop a comprehensive database of information, and provide firms with incentives for pre‐market voluntary submissions (Diamond, 2008; Ventola, 2012). With better data, the regulator is in a better position to assess the submitted nanoproducts, thereby increasing the efficiency of its procedures. In essence, regulatory bodies must shift from their approach considering nanoparticles as small versions of larger molecules to one where they recognize their fundamental different properties (Bawa et al., 2016; Fischer, 2018; Ventola, 2012).

Alternatively, the public funder must take action. Early initiatives in nanomedicine and nanobiotechnology had already identified the reticence of pharmaceutical firms in embracing novel and risky‐seeming nanomedicinal research (Jackman, Lee, & Cho, 2016; Keelan, Leong, Ho, & Iyer, 2015; Lenoir & Herron, 2015). The National Cancer Institute illustratively launched the Alliance for Nanotechnology in Cancer in 2004 to ignite nano‐scale products for cancer diagnosis, prevention, and treatment. The most promising research avenues developed by the Alliance are then handed off to private sector partners for effective clinical translation and commercialization.^3^ The Alliance has funneled taxpayer funds into early‐stage research, spawning a number of advances in nanoparticle vaccines (PRINT particle design by Liquidia Technologies), in‐vitro blood diagnostics (T2Hemostasis devices which utilize the proprietary T2 Magnetic Resonance by T2 Biosystems), as well as drug targeting and delivery more broadly (AxioCore nanofiber drug delivery by Arsenal Vascular; Lenoir & Herron, 2015). Such targeted initiatives have the potential to lead to patents in novel but uncertain fields, pushing boundaries for‐profit firms would not necessarily be willing to cross.

## CONFLICT OF INTEREST

The authors have declared no conflicts of interest for this article.

## RELATED WIREs ARTICLES

https://doi.org/10.1002/wnan.1416

https://doi.org/10.1002/wnan.1527

https://doi.org/10.1002/wnan.1465

## ENDNOTES

1 US Food and Drug Administration, Drug Products, Including Biological Products, that Contain Nanomaterials, Guidance for Industry, December 2017.

2 European Patent Office. Nanotechnology and Patents. 2013.

3 National Cancer Institute, Cancer Nanotechnology Plan 2015.
